# Supplementary material for: Coumarinolignoid and Indole Alkaloids from the Roots of the Hybrid Plant Citrus × paradisi Macfad (Rutaceae)
Source: Molecules. 2023 Jan 20;28(3):1078. doi: 10.3390/molecules28031078 (PMC9921193; doi:10.3390/molecules28031078)
Supplement: Supplementary file 1 [file molecules-28-01078-s001.zip › molecules-2136584-supplementary.pdf]

Coumarinolignoid and Indole Alkaloids from the Roots of the Hybrid Plant Citrus x paradisi Macfad. (Rutaceae)

Fanny-Aimée Essombe Malolo [1], Ariane Kenmogne Kouam [1], Judith Caroline Ngo Nyobe [2], Lidwine Ngah [2], Marcel Frese [3], Jean Claude Ndom [1], Moses K. Langat [4], Bruno Ndjakou Lenta [3,5], Dulcie Mulholland [6], Norbert Sewald [3,*] and Jean Duplex Wansi[1,3,*]

1 Department of Chemistry, Faculty of Sciences, University of Douala, Douala P.O. Box 24157, Cameroon; em-fay1@yahoo.fr (F.-A.E.M.); arydolls@gmail.com (A.D.K.K.); ndomjefr@yahoo.fr (J.C.N.)
2 Department of Pharmaceutical Sciences, Faculty of Medicine and Pharmaceutical Sciences, University of Douala, Douala P.O. Box 2701, Cameroon; njudithcaroline@yahoo.fr (J.C.N.N.); lidwingah@yahoo.fr (L.N.)
3 Organic and Bioorganic Chemistry, Department of Chemistry, Bielefeld University, 33501 Bielefeld, Germany; marcel.frese@uni-bielefeld.de (M.F.); lentabruno@yahoo.fr (B.N.L.)
4 Royal Botanic Gardens, Kew, Kew Green, Richmond TW9 3AE, UK; m.langat@kew.org
5 Department of Chemistry, Higher Teacher Training College, University of Yaoundé 1, Yaoundé P.O. Box 47, Cameroon
6 Natural Products Research Group, Department of Chemistry, Faculty of Engineering and Physical Sciences, University of Surrey, Guildford GU2 7XH, UK, d.mulholland@surrey.ac.uk
* Correspondence: norbert.sewald@uni-bielefeld.de (N.S.); jdwansi@yahoo.fr or jean.wansi@uni-bielefeld.de (J.D.W.)

Abstract: Phytochemical investigation of the roots of *Citrus x paradisi* Macfad. (Rutaceae) led to the isolation of two new compounds, namely 1-formyl-5-hydroxy-*N*-methylindolin-1-ium (**1**) and decyloxycleomiscosin D (**2**), along with ten known compounds 1,1-dimethylpyrrolidin-1-ium-2-carboxylate (**3**), furan-2,3-diol (**4**), 5-methoxyseselin (**5**), umbelliferone (**6**), scopoletin (**7**), citracridone I (**8**), citracridone II (**9**), citracridone III (**10**), limonin (**11**) and lupeol (**12**). Structures were determined by comprehensive spectroscopic analysis of 1D and 2D NMR and EI- and ESI-MS as well as comparison with published data. Notably, compounds **3** and **4** are reported here from the genus *Citrus* for the first time. In addition, the MeOH extract of the roots and compounds **1** - **7** were screened against the human adenocarcinoma alveolar basal epithelial cell line A549 and the Caucasian prostate adenocarcinoma cell line PC3 using the MTT assay. While the extract showed significant activity with IC_{50} values of 35.2 and 38.1 µg/mL, respectively, compounds **1** - **7** gave weak activity with IC_{50} values of 99.2 to 250.2 µM and 99.5 to 192.7 µM, respectively.

Keywords: *Citrus x paradisi*; coumarinolignoid; indole alkaloids; chemotaxonomy; cytotoxicity

COMPOUND 1

Figures	**Spectral**	**Pages**
Figure S1	ESIMS	3
Figure S2	^{1}H NMR	4
Figure S3	COSY	5
Figure S4	^{13}C NMR	6
Figure S5	DEPT	7
Figure S6	HSQC	8
Figure S7	HMBC	9
Figure S8	NOESY	10

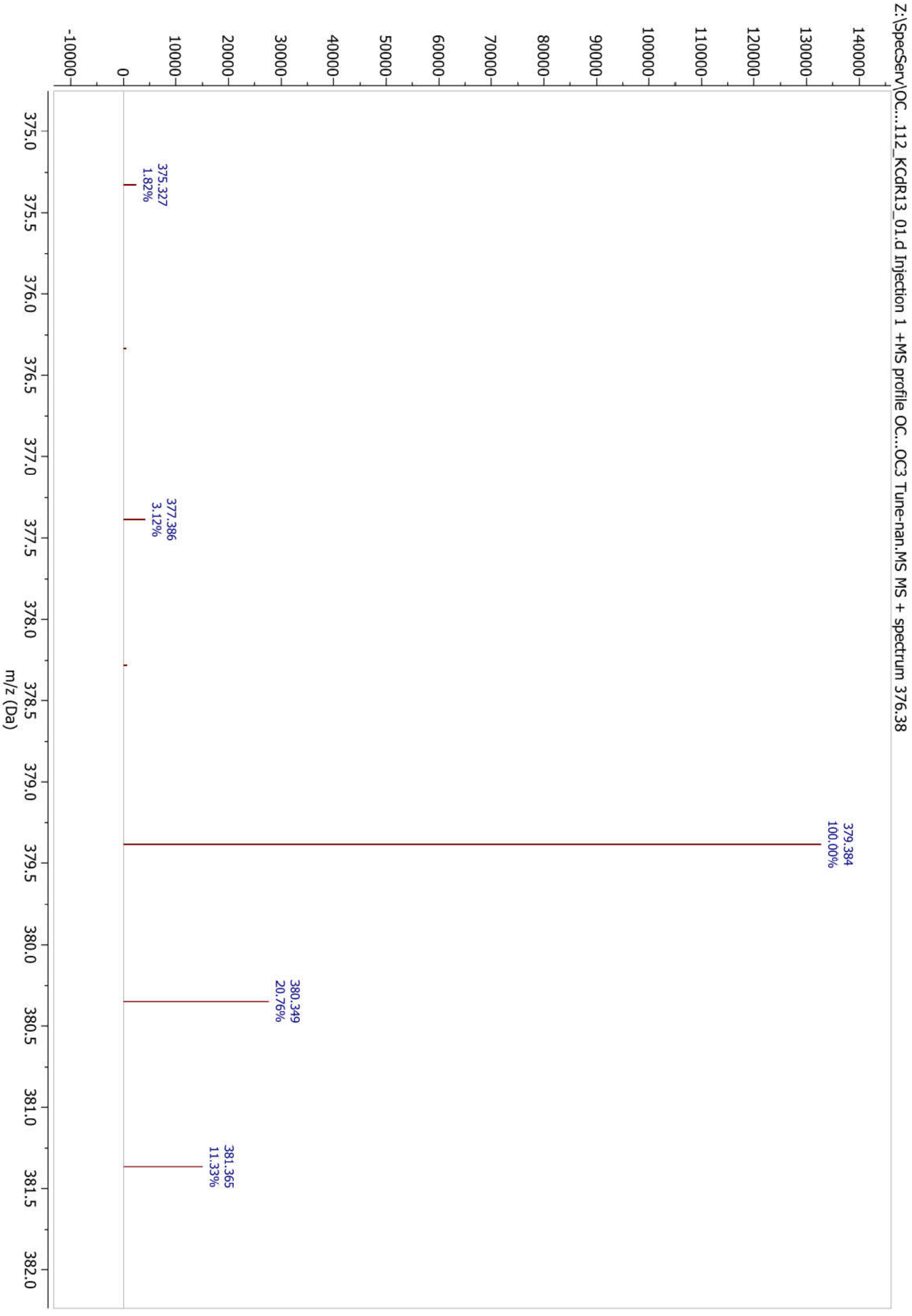

Figure S1: ESIMS of 1

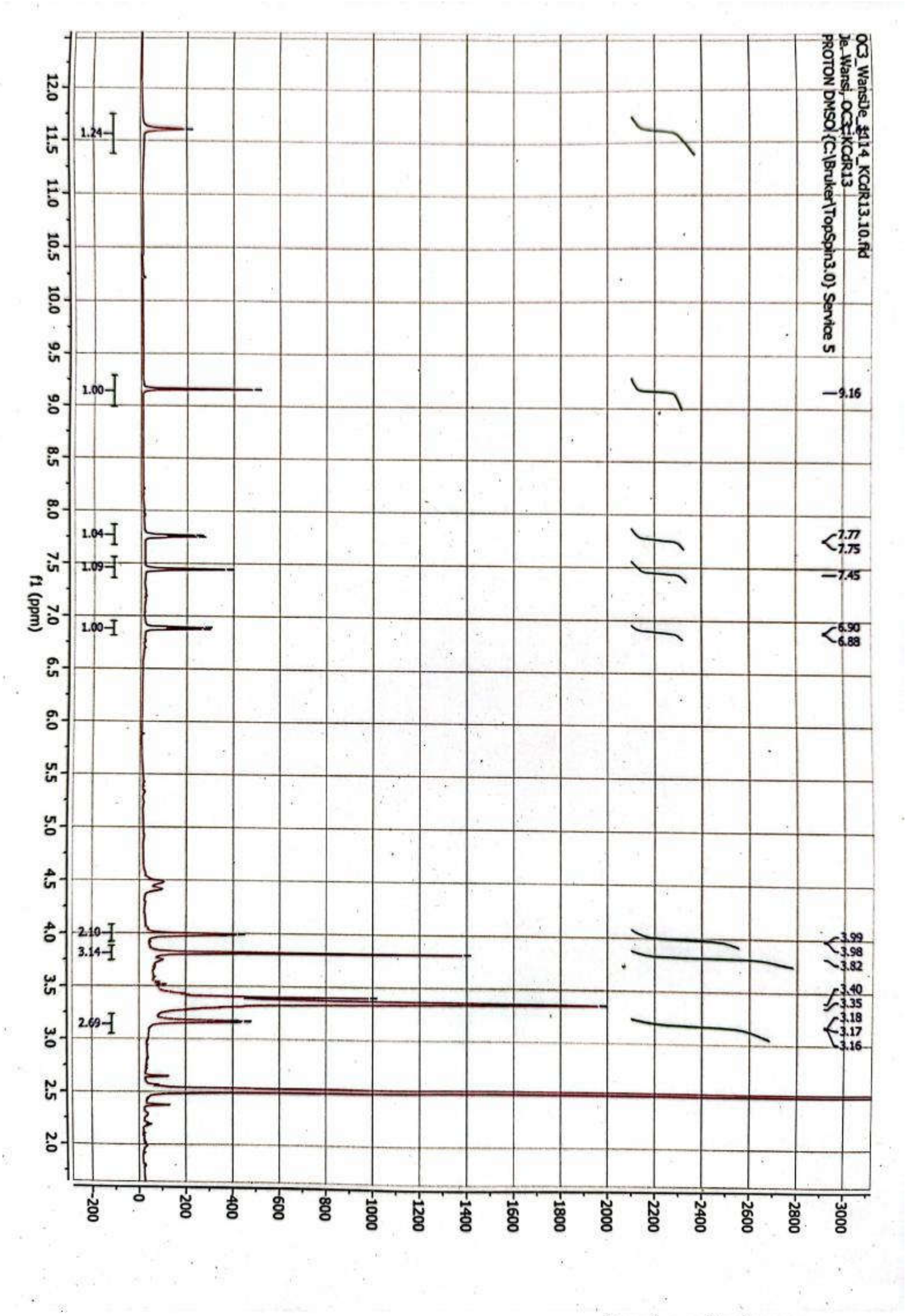

Scanné avec CamScanner

Figure S2: ^{1}H NMR of 1

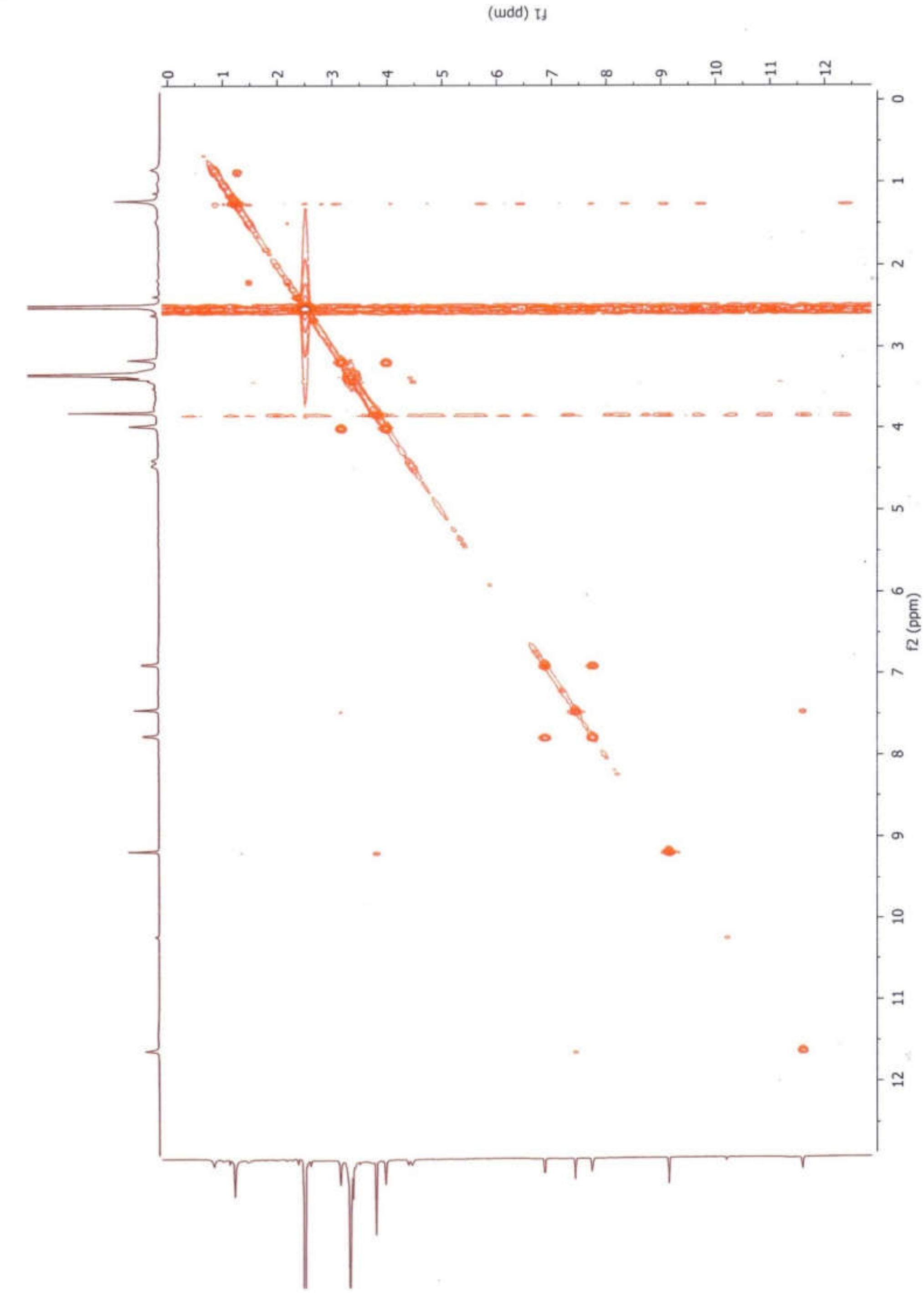

Figure S3: COSY of 1

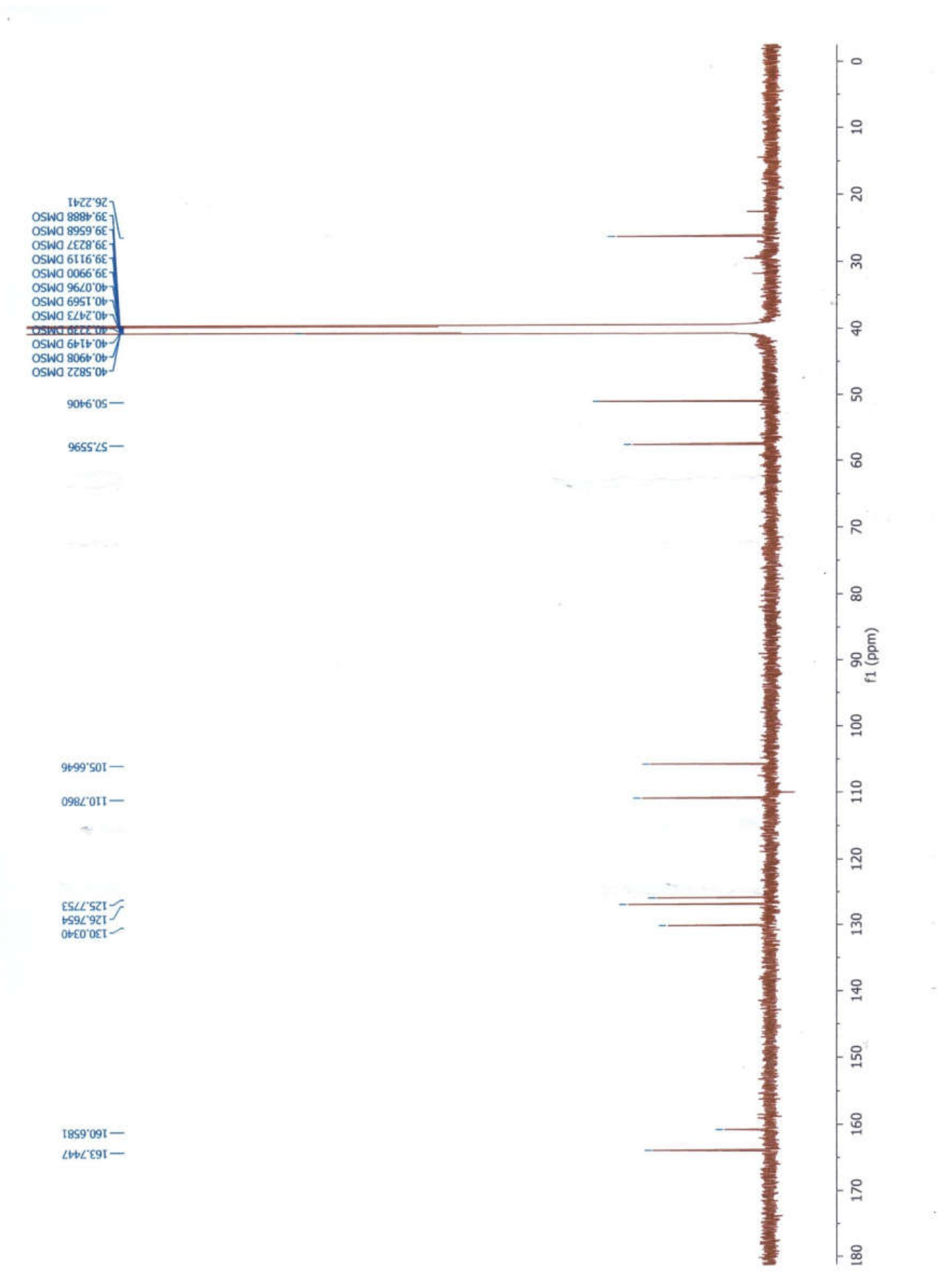

Figure S4: ^{13}C-NMR of 1

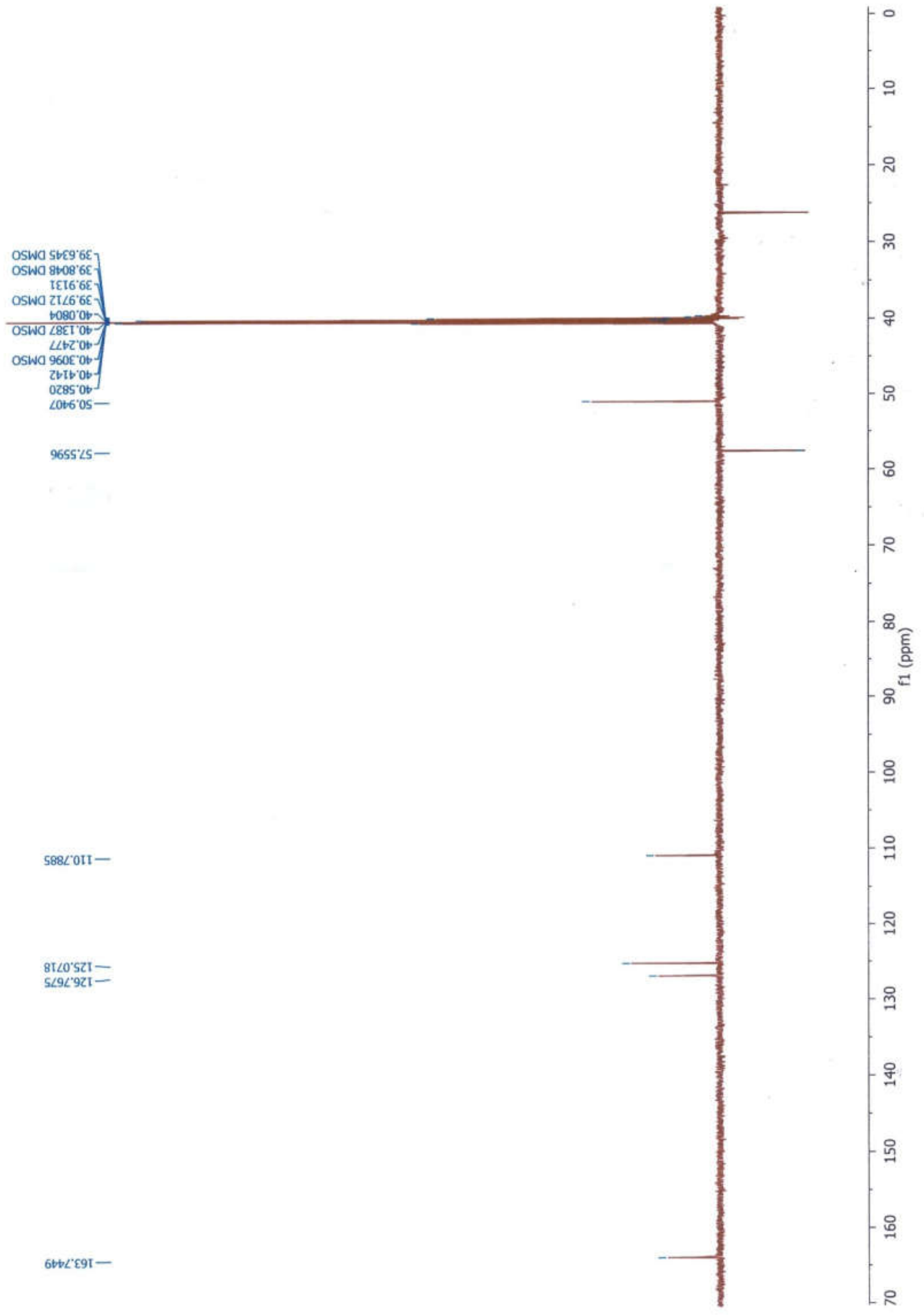

Figure S5: DEPT of 1

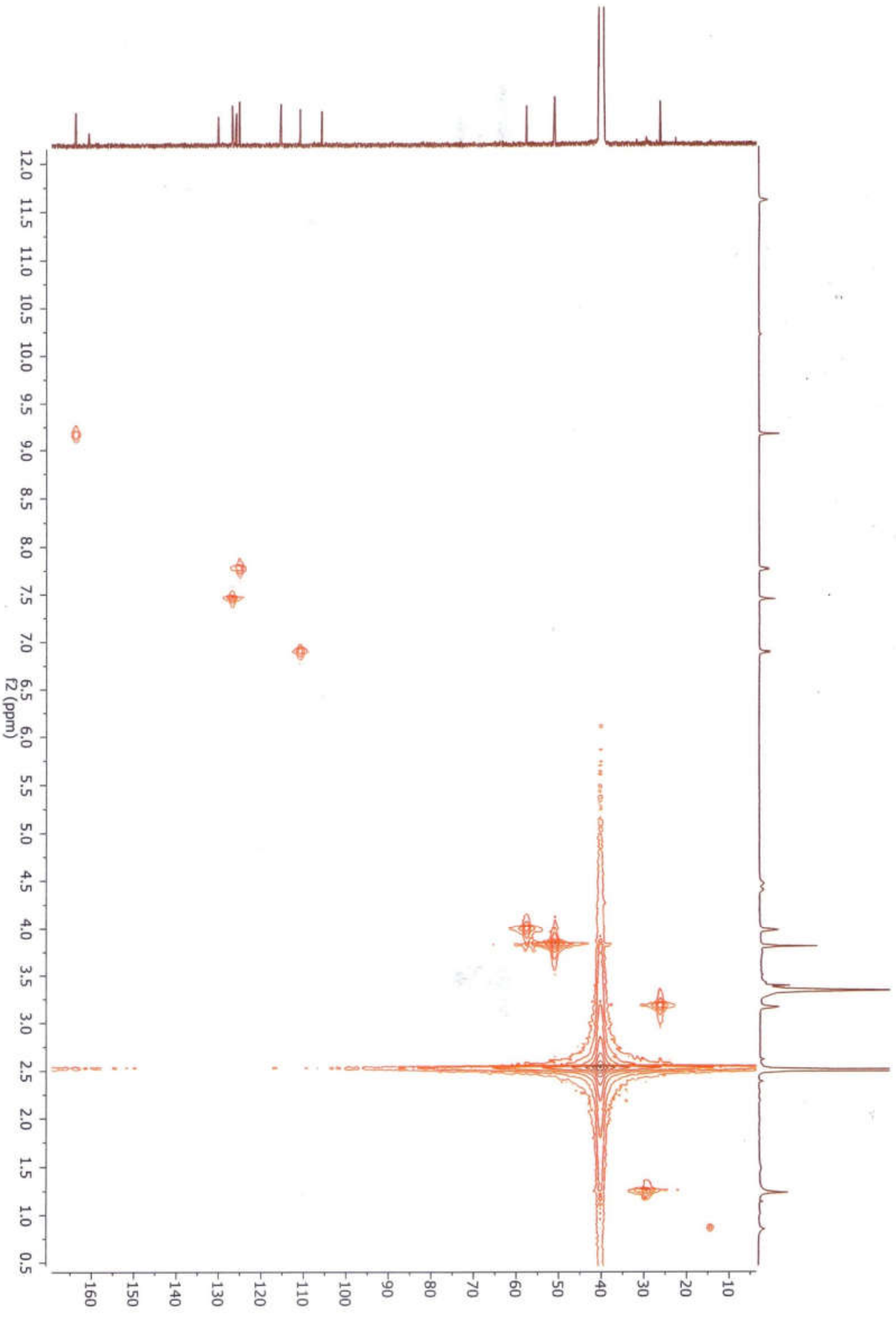

Figure S6: HSQC of 1

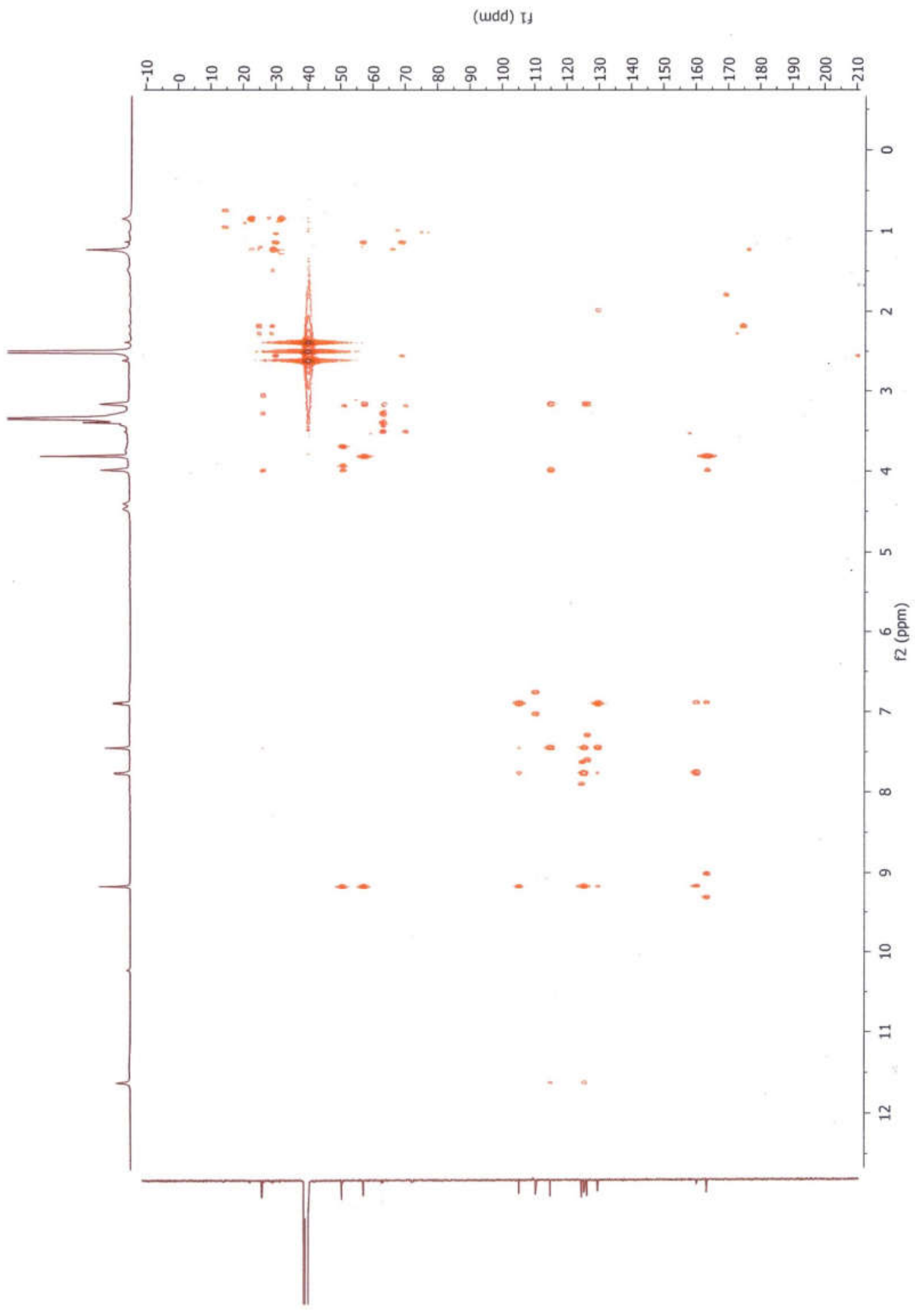

Figure S7: HMBC of 1

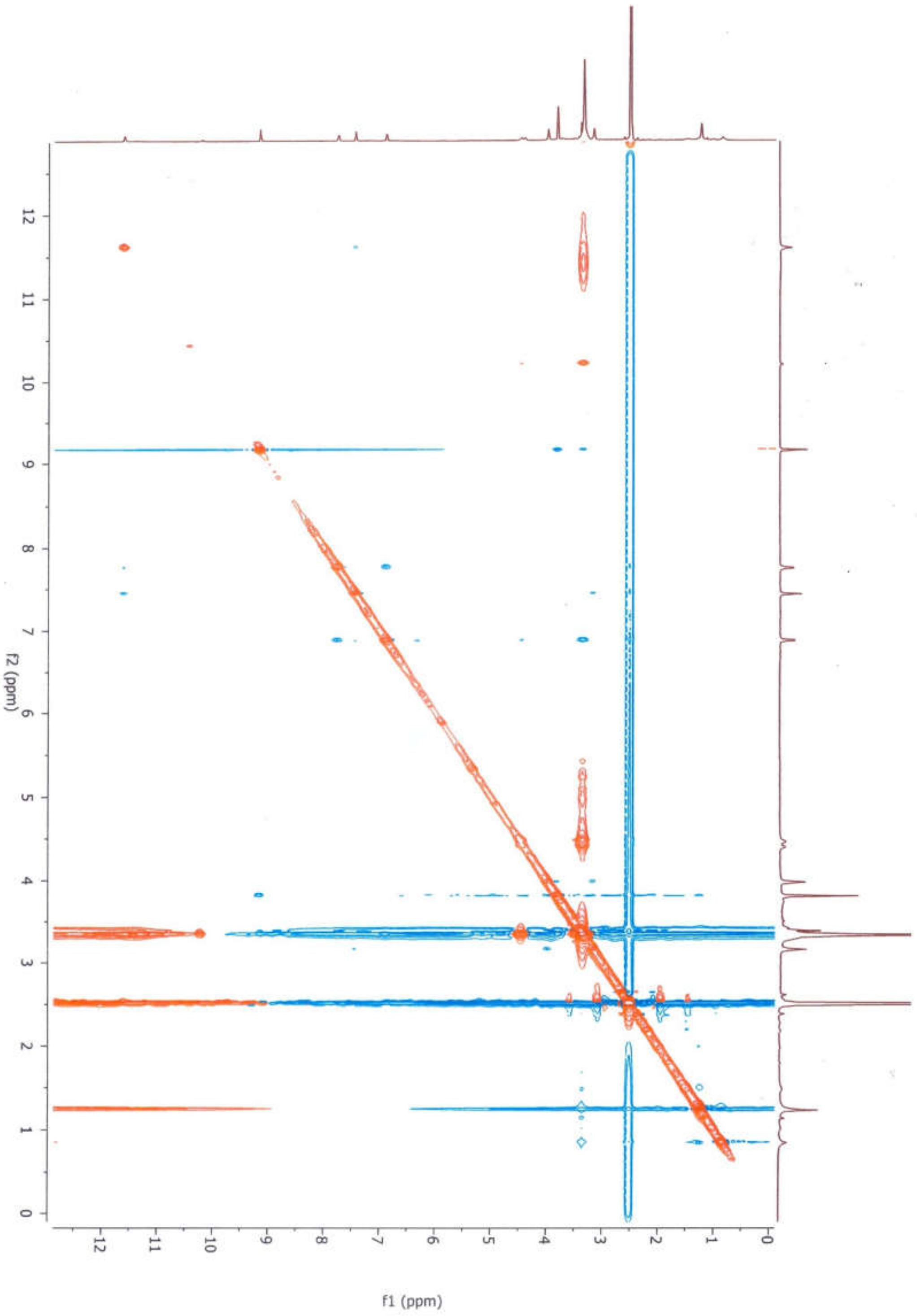

Figure S8: NOESY of 1

COMPOUND 2

**Figures**	**Spectral**	**Pages**
Figure S9	ESIMS	12
Figure S10	^{1}H NMR	13
Figure S11	^{13}C NMR	14
Figure S12	DEPT	15
Figure S13	COSY	16
Figure S14	HSQC	17
Figure S15	HMBC	18
Figure S16	NOESY	19
**Acid hydrolysis to obtain nonanol**		
Figure S17	EIMS of nonanol	20
Figure S18	^{1}H NMR of nonanol	21

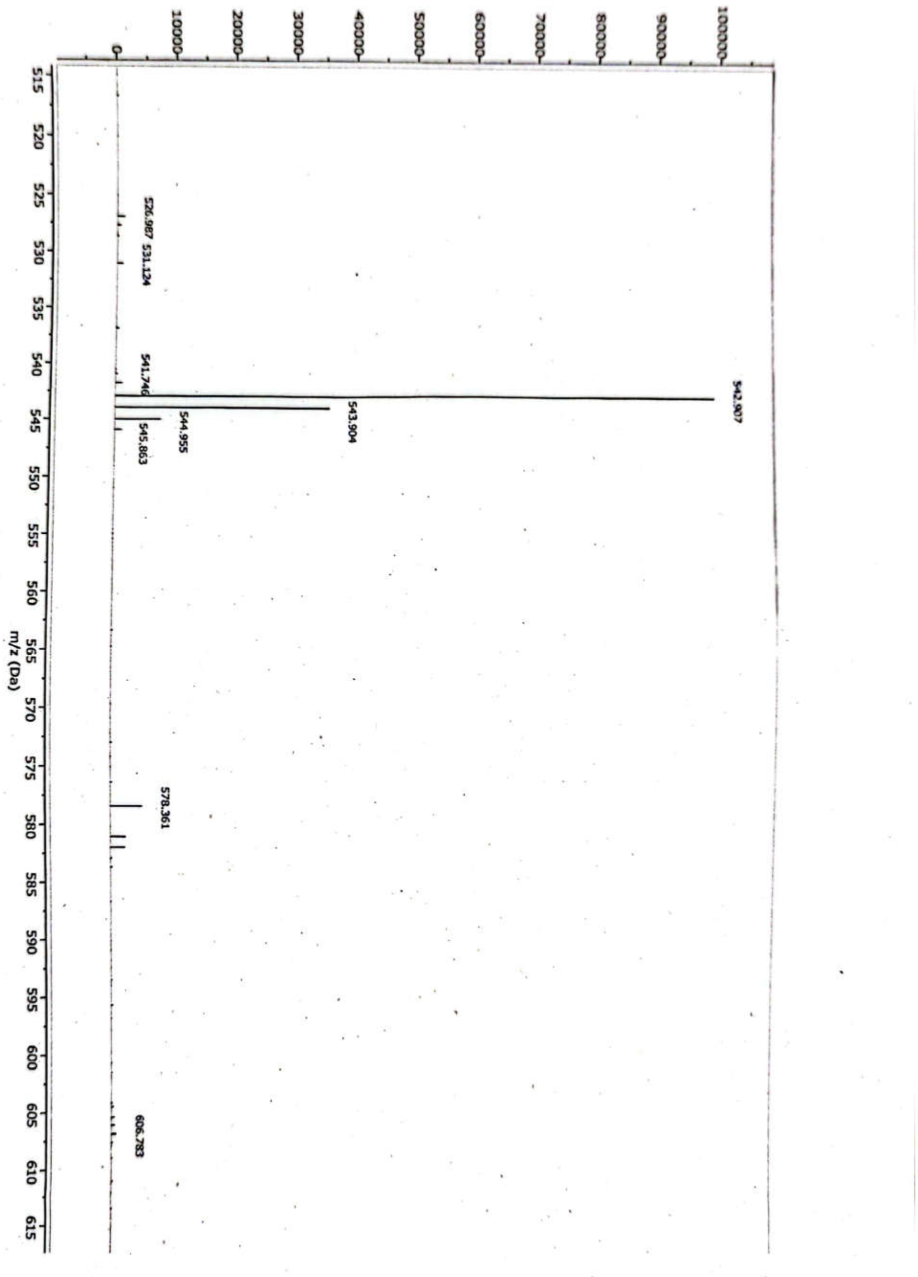

Scanné avec CamScanner

Figure S9: ESIMS of 2

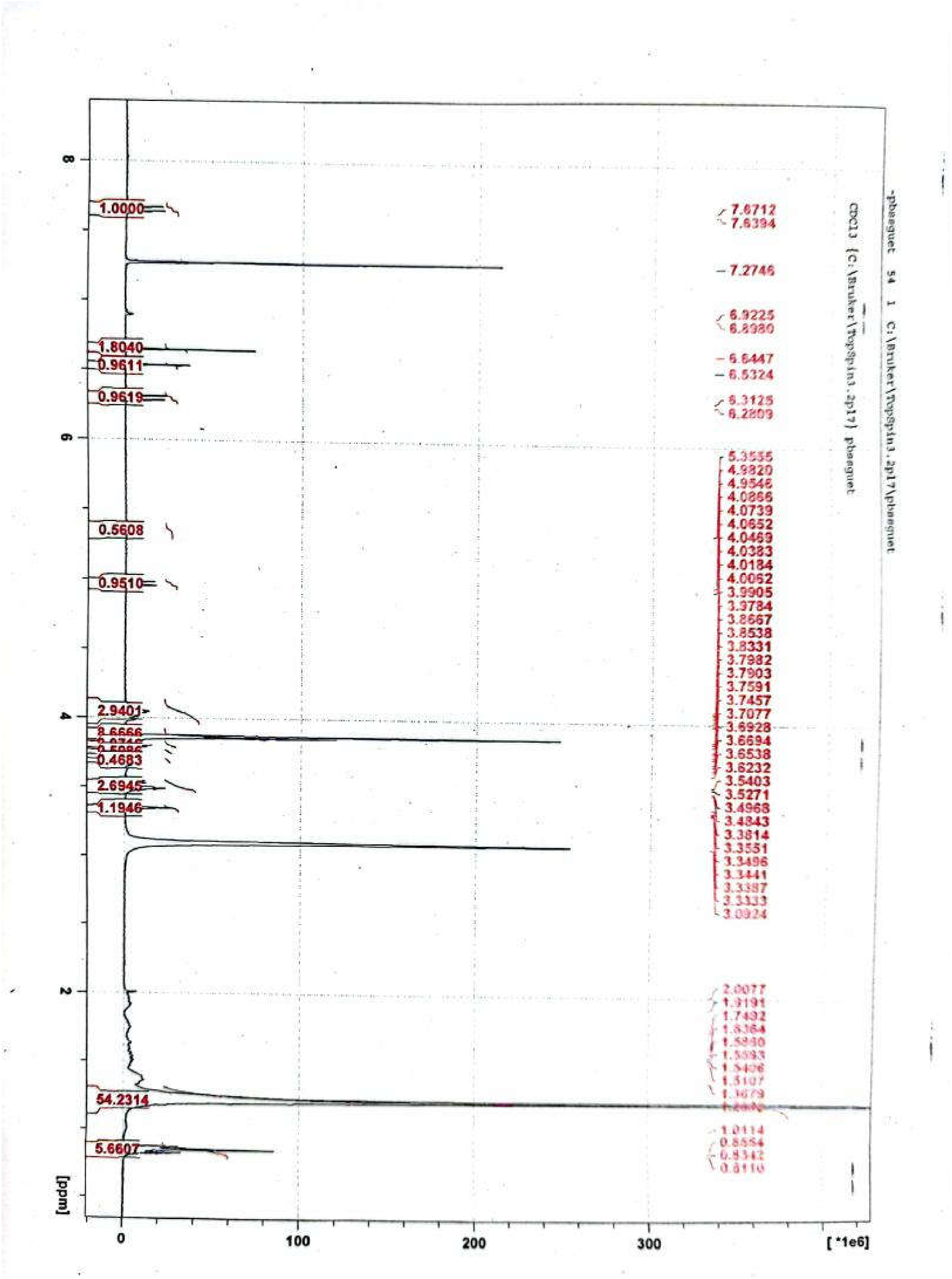

Scanné avec CamScanner

Figure S10: ^{1}H NMR of 2

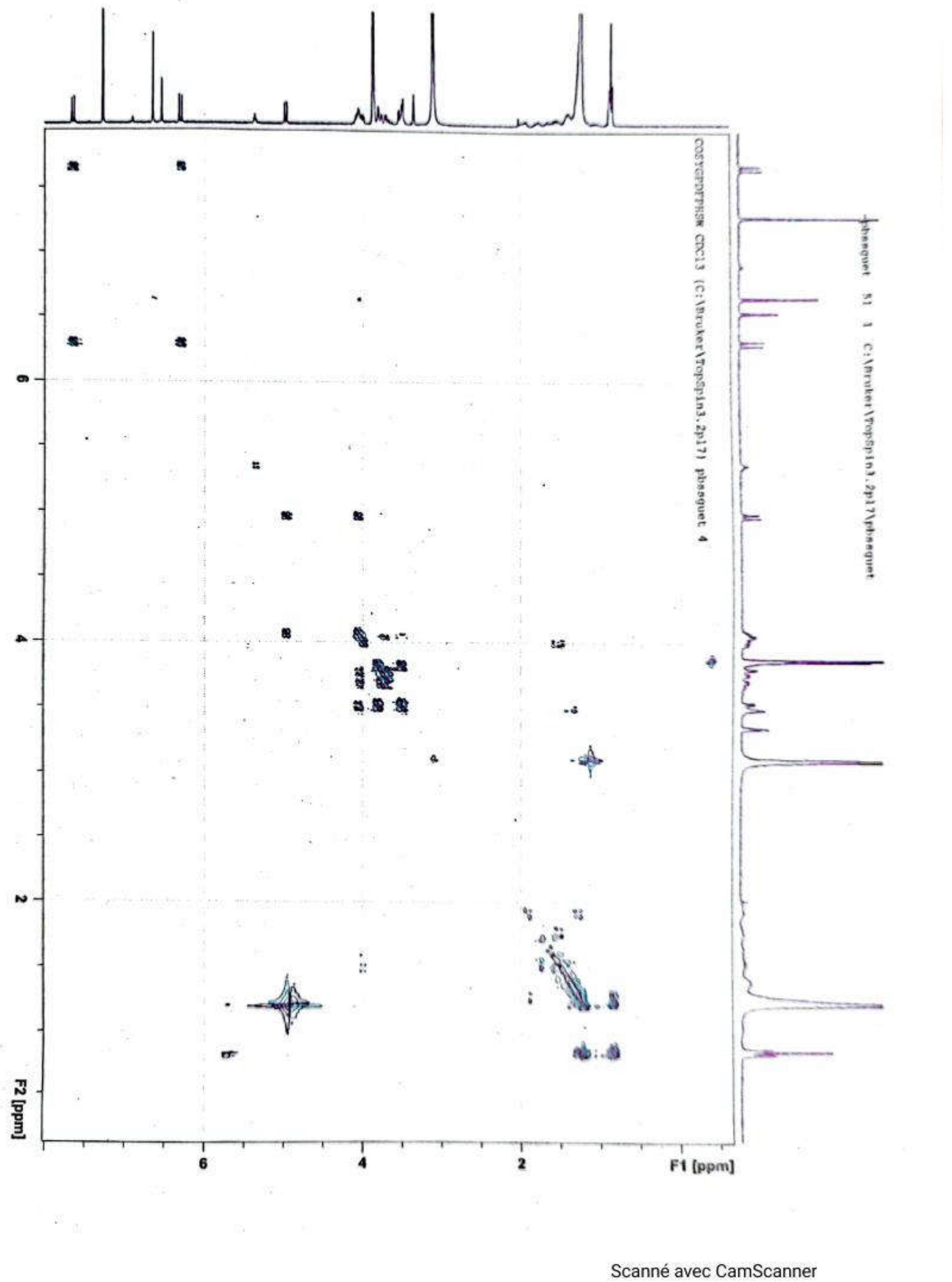

Scanné avec CamScanner

Figure S11: COSY of 2

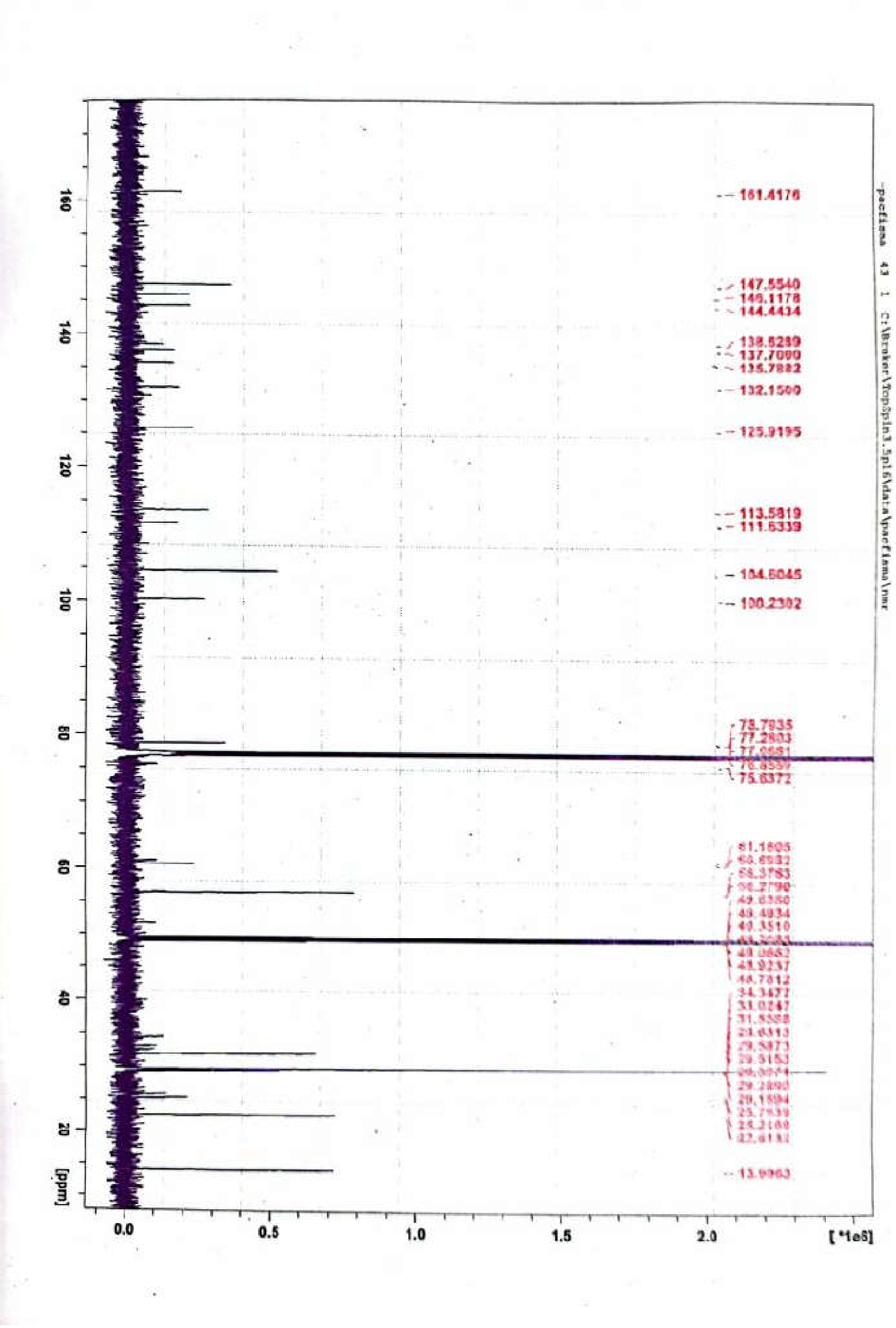

Figure S12: ^{13}C NMR of 2

Scanné avec CamScanner

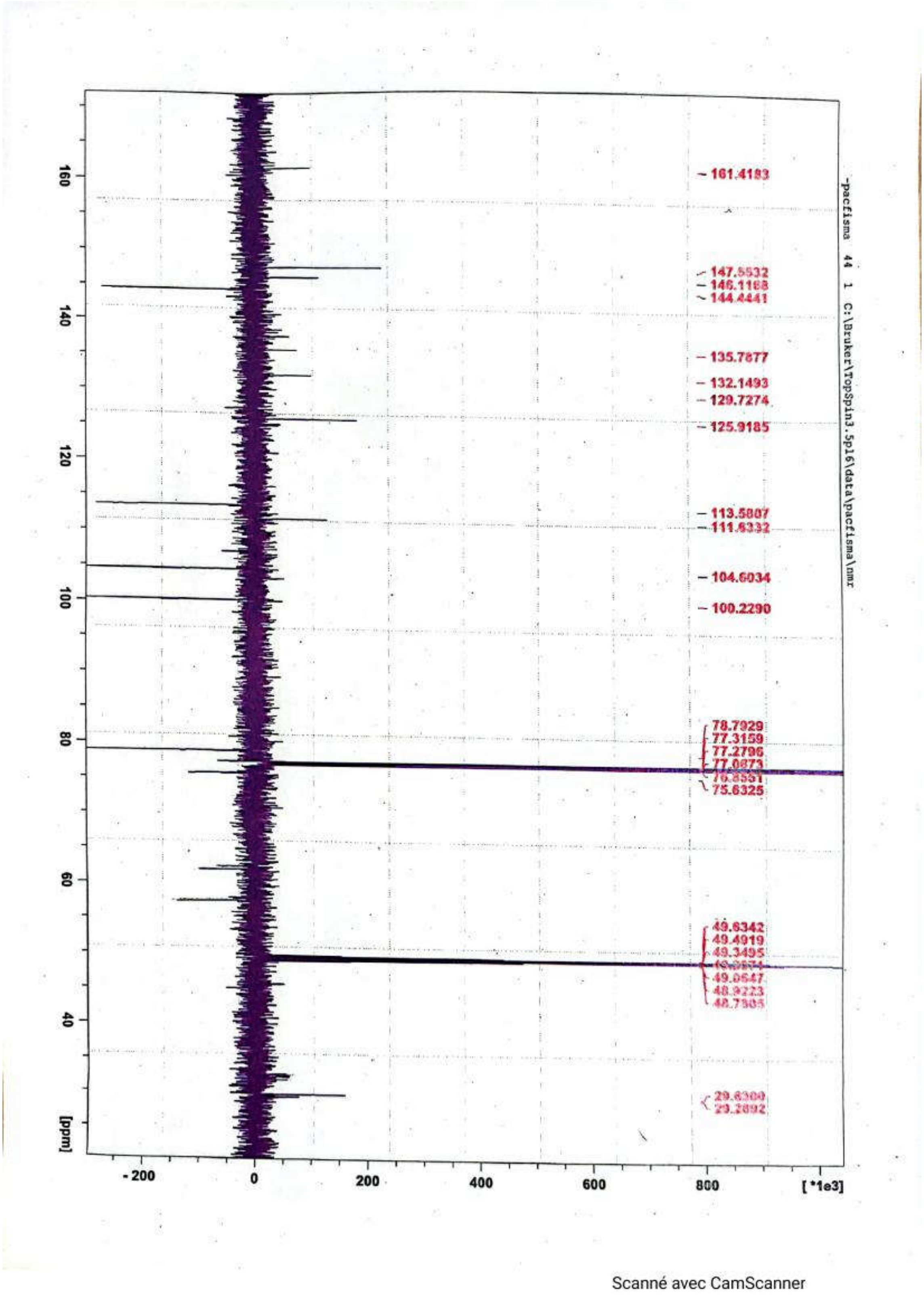

Figure S13: ^{13}C (J.mod) of 2

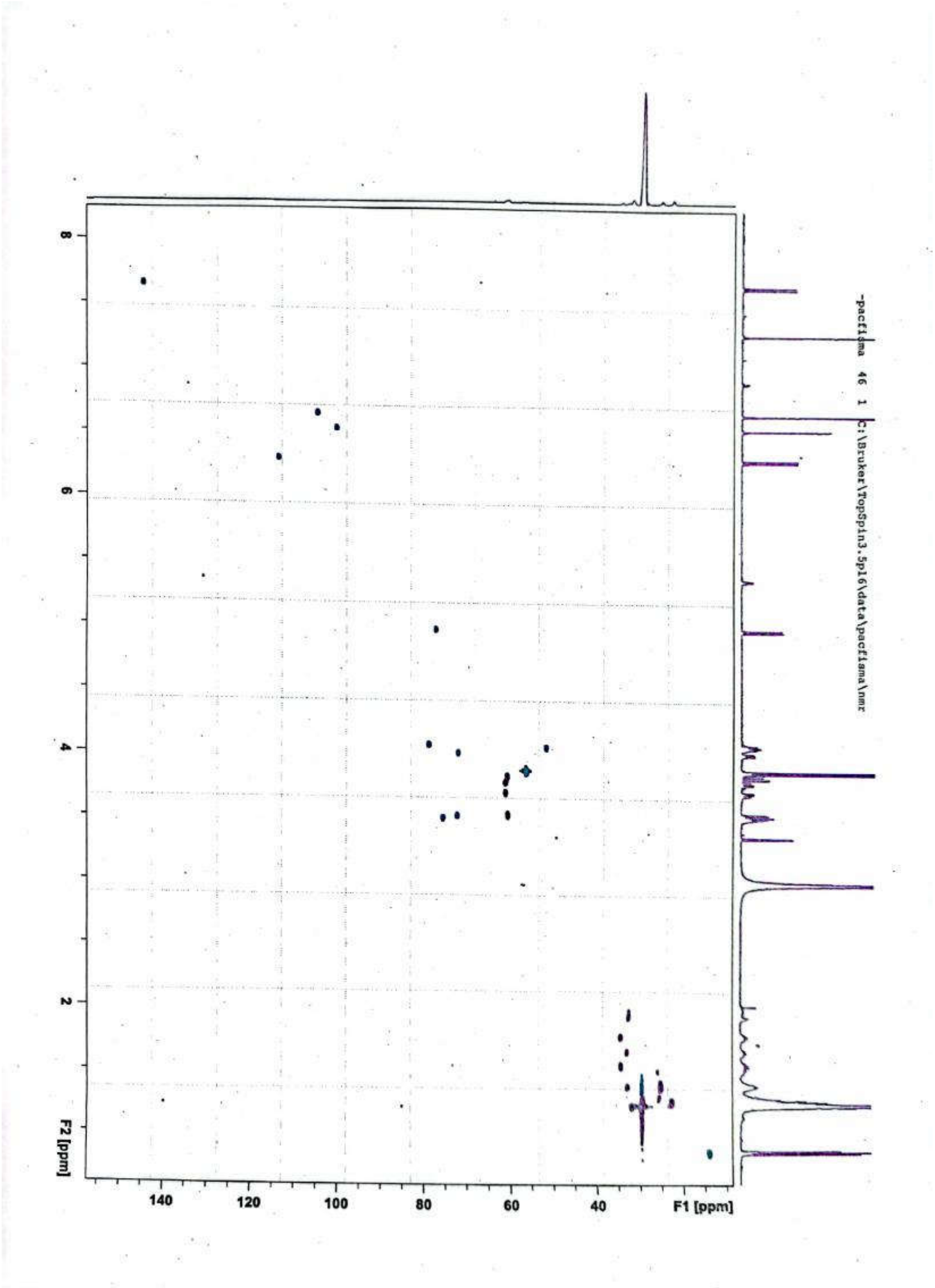

Scanné avec CamScanner

Figure S15: HSQC of 2

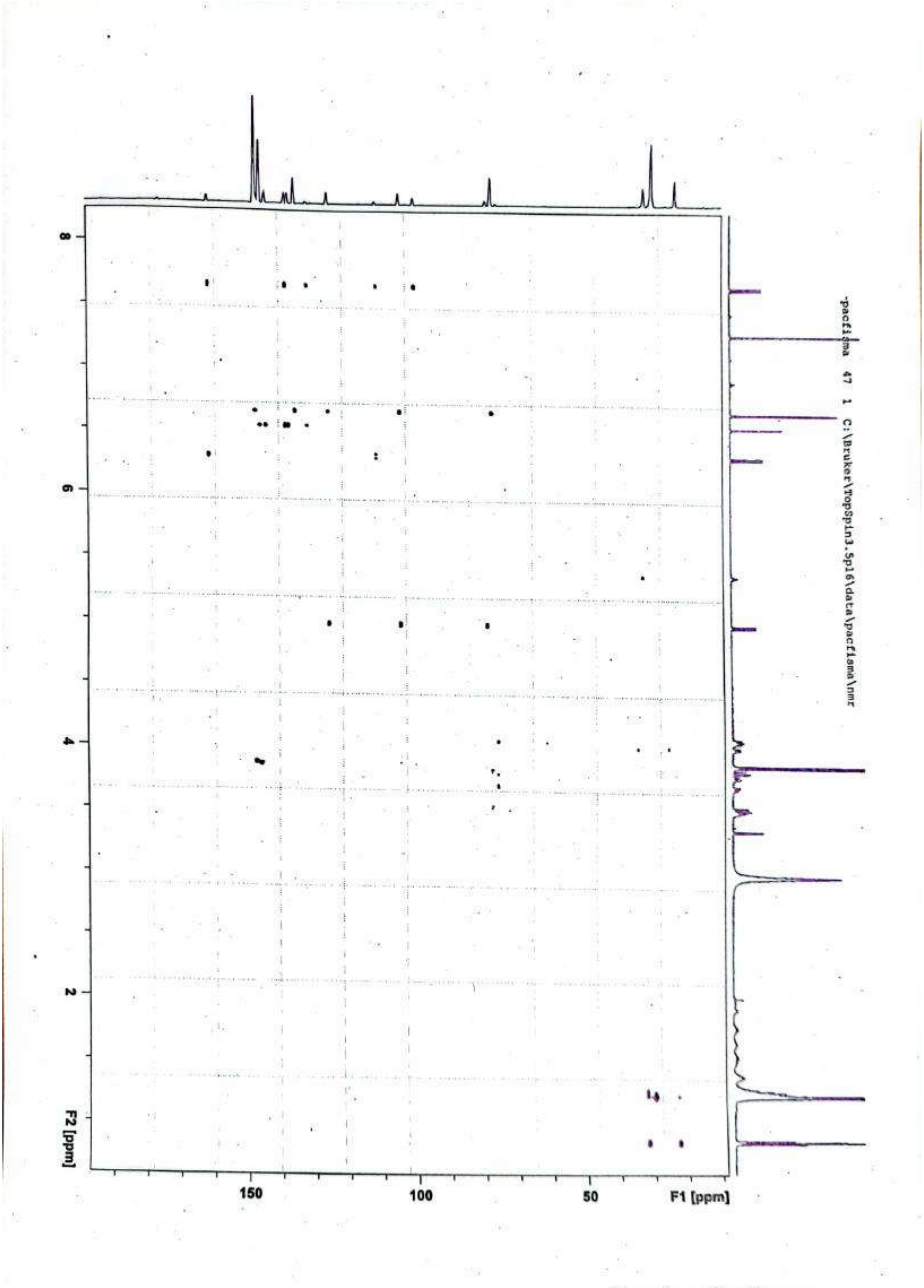

Scanné avec CamScanner

Figure S15: HMBC of 2

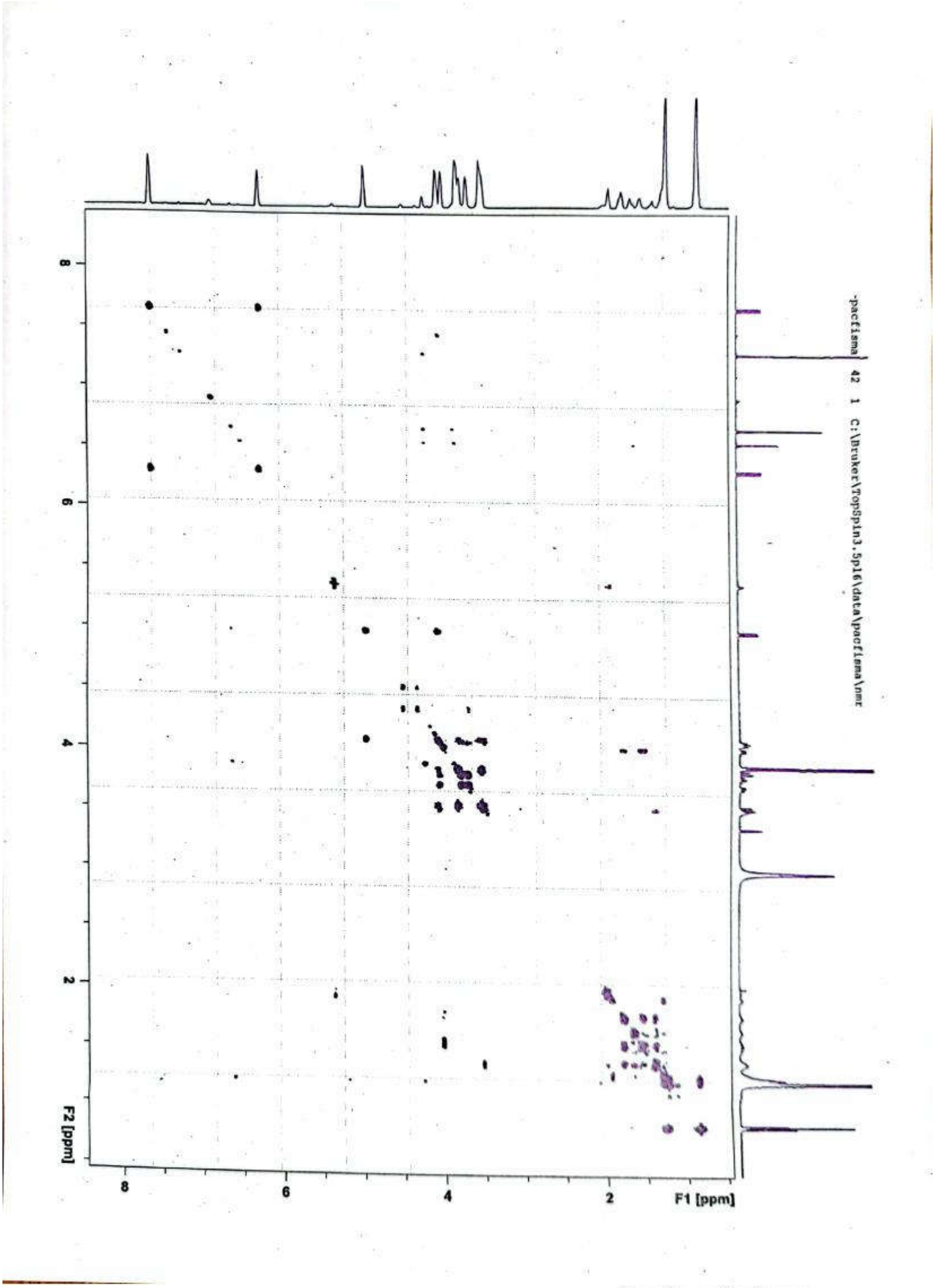

Scanné avec CamScanner

Figure S16: NOESY of 2

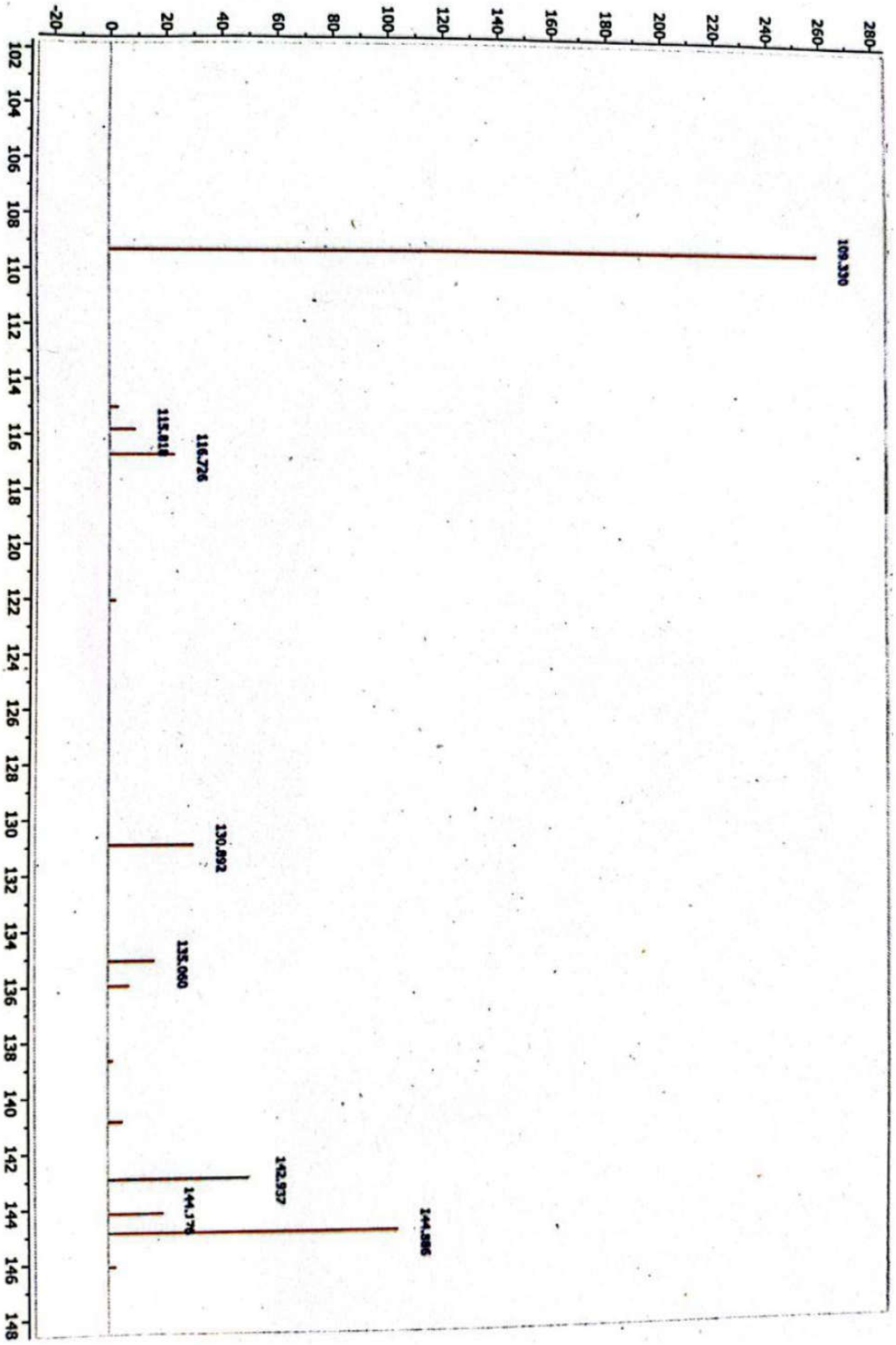

Figure S17: EIMS of nonanol

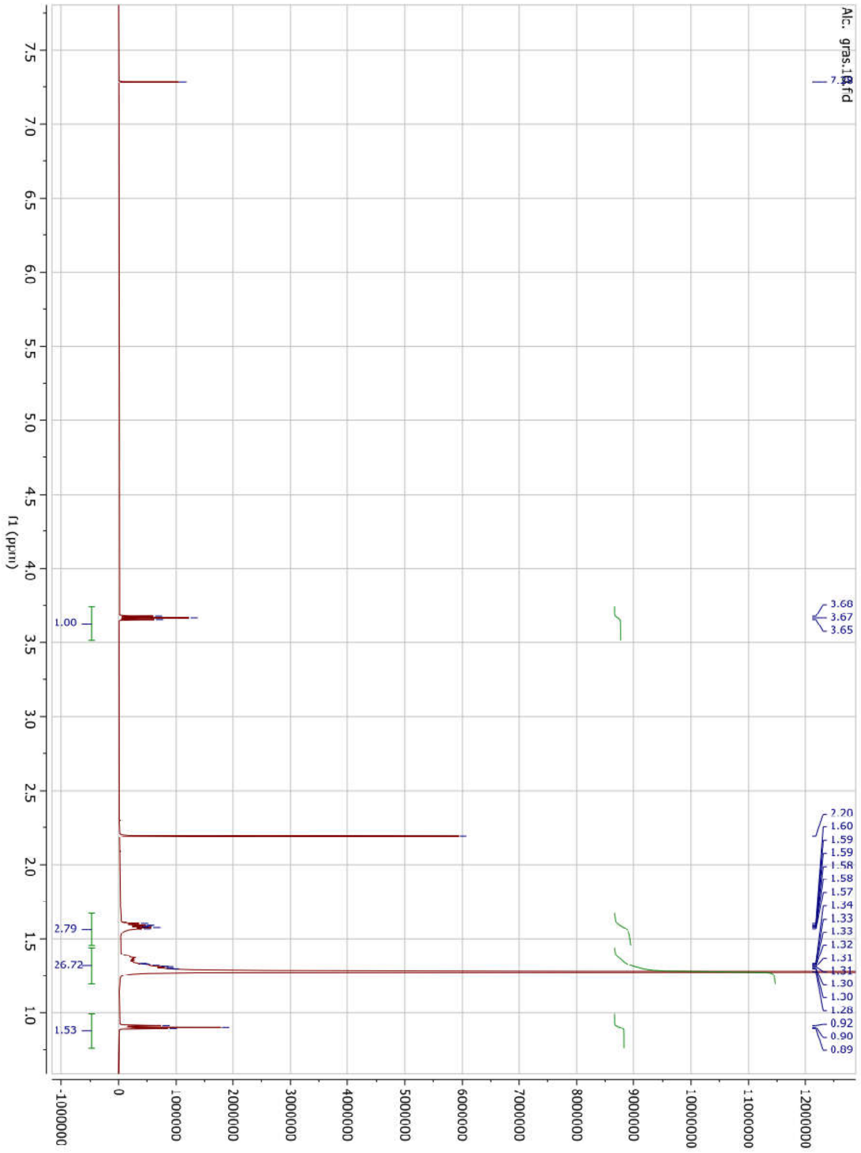

Figure S18: ^{1}H NMR of nonanol
